# Simulations of Transients in a Four-Pole Magnetic Bearing with Permanent Magnets

**DOI:** 10.3390/s24051402

**Published:** 2024-02-22

**Authors:** Dawid Wajnert, Bronisław Tomczuk

**Affiliations:** Department of Electrical Engineering and Mechatronics, Opole University of Technology, PL-45758 Opole, Poland; b.tomczuk@po.edu.pl

**Keywords:** radial magnetic bearing, permanent magnets, transient simulation model

## Abstract

This paper presents the design of and transient time simulations for a four-pole magnetic bearing with permanent magnets. The usage of permanent magnets reduces the consumption of electric energy in comparison to a traditional active magnetic bearing. Permanent magnets are installed in the yoke of the stator core to limit the cross-coupling of the magnetic flux generated by the windings. The first part of this paper presents the design of the magnetic bearing and its finite-element model, while the second part describes the field-circuit indirectly coupled finite-element model for the transient time simulation. The presented simulation model was used to calculate the transient response for the rotor lifting from the starting position, the step change in the rotor position and the change in the rotor position under an external impact force applied along the *y*-axis.

## 1. Introduction

A magnetic bearing is a special type of electric machine that uses a magnetic field to levitate a rotor without mechanical contact. Therefore, magnetic bearings have unique properties like a very high rotational speed, operation without lubrication, a long lifetime, operation in a vacuum and operation in a clean/harsh environment [1]. These advantages of magnetic bearings make them usable in industrial applications like flywheel energy storage, electrospindles, high-speed motors, blowers, blood pumps and turbo generators.

Magnetic bearings can be classified into three various types, such as active magnetic bearings [2,3], passive magnetic bearings [4] and active magnetic bearings with permanent magnets, which are also called hybrid ones [5]. Active magnetic bearings require a pre-magnetized magnetic circuit for proper operation. Therefore, the so-called bias flux is produced by the constant current flowing through the excitation windings [1]. The magnetic force generated by the magnetic bearing is controlled by the control flux produced by the control current. Passive magnetic bearings use a combination of an attractive and repulsive force of permanent magnets for the levitation of the rotor [6]. Unfortunately, according to Earnshaw’s theorem, it is impossible to build a stable passive bearing for all axes. The working principle of magnetic bearings with permanent magnets is very similar to that of active magnetic bearings with one distinction: permanent magnets are used to produce the bias flux while the control current produces the control flux only. The usage of permanent magnets increases the efficiency of magnetic bearings; therefore, in recent years, an increase in research activity concerning magnetic bearings with permanent magnets can be seen [7,8,9,10].

Magnetic bearings with permanent magnets require a control system for the stable levitation of the rotor. Therefore, to determine the dynamic response of the designed magnetic bearing, a proper dynamic simulation model is necessary. There are various simulation models dedicated to transient simulations for magnetic bearings. The straightforward simulation model is based on partial differential equations (PDEs) that describe the motion of the rotor, and the magnetic bearing is simulated as a current-controlled device with two parameters: current and position stiffness [11]. Additionally, this model can take into account the voltage drop across windings.

The second type of dynamic simulation model is based on PDEs that describe the motion of the rotor and the voltage drop across windings, while the magnetic force and flux linkage are calculated from the magnetic equivalent circuit (MEC) [12,13]. The MEC of a magnetic bearing can incorporate the nonlinear characteristics of the magnetic material as well as leakage and fringing fluxes [14]. There are two variants of this dynamic simulation model. For the first one—named a field-circuit directly coupled MEC—the equations for the magnetic equivalent circuit are solved at every simulation step of the solution for the PDEs [13]. For the second variant—named a field-circuit indirectly coupled MEC—quantities like magnetic force and flux linkages are calculated beforehand, and they are included in the simulation model as look-up tables.

The last type of dynamic simulation model is based on PDEs that describe the motion of the rotor and the voltage drop across windings, while the magnetic force and flux linkage are calculated from the finite-element model (FEM). Both 2D and 3D models with nonlinear B–H characteristics can be implemented to simulate the magnetic field distribution. Also, we can distinguish two variants of this dynamic simulation model. For the first variant—named a field-circuit directly coupled FEM and often called a time-stepping FEM—the equations for the magnetic field distributions are integrated into the equations of the rotor motion and the voltage drop across windings. Then, the whole system of equations is solved at every simulation step; therefore, the simulation time is very long [15,16]. For the second variant of this simulation model—named a field-circuit indirectly coupled FEM—quantities like the magnetic force and flux linkages are calculated earlier, and they are incorporated into the simulation model as look-up tables [17].

The most accurate results are obtained for dynamic simulation models based on the finite-element method. Unfortunately, the field-circuit directly coupled FEM is very time-consuming; therefore, the second variant of this simulation model has gained popularity [17,18].

The aim of our paper was not only the proposition of a new geometry of a four-pole magnetic bearing with four permanent magnets installed in the stator yoke but also a simulation of its dynamics. The application of the permanent magnets reduces the consumption of the electric energy, and placing them in the stator yoke limits the cross-coupling of the magnetic fluxes between windings.

The first part of the paper presents the construction, principle of operation and magnetic field simulation of the four-pole magnetic bearing. The second part describes the simulation model dedicated to the analysis of transients. Our algorithm is based on the field-circuit indirectly coupled FEM. The three-dimensional FEM was used to determine the magnetic flux distributions, calculate the rated parameters and obtain the lookup tables required for the dynamic simulation model. Based on the proposed dynamic simulation model, transient responses like the rotor lifting from the starting position, the step change in the rotor position and the change in the rotor position under an external impact force applied along the *y*-axis were simulated.

## 2. Structure of the Magnetic Bearing with Permanent Magnets

**Figure 1:**
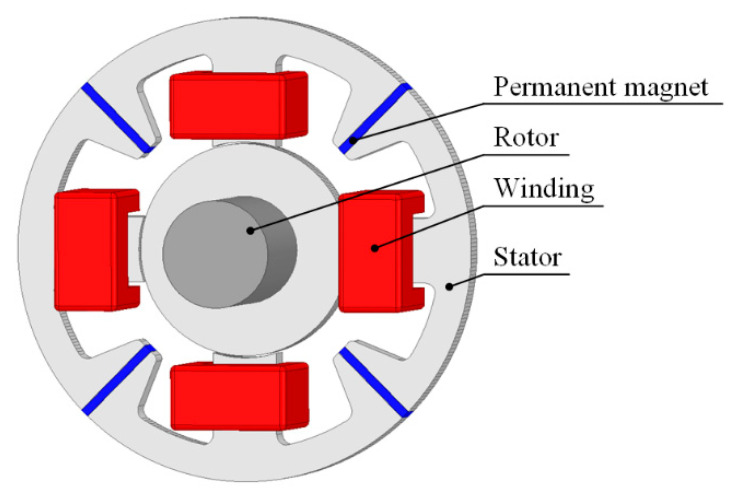
Structure of the magnetic bearing with permanent magnets.

**Figure 2:**
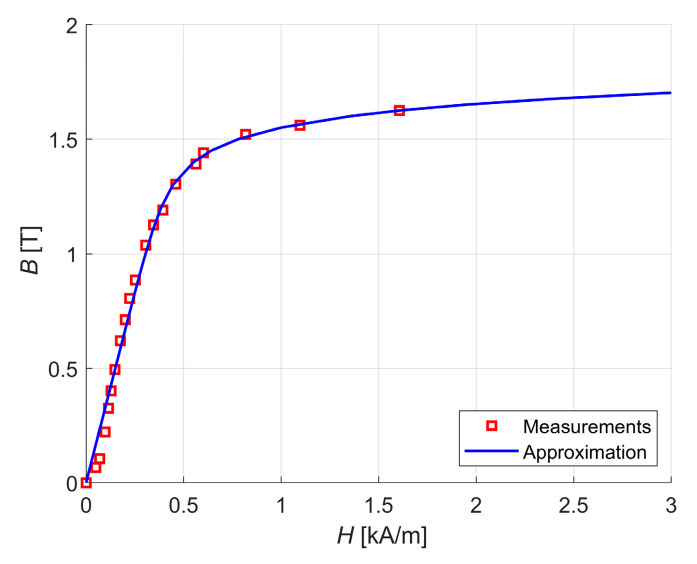
The B–H curve of the M400-50A silicon steel.

Figure 1 presents the geometry of the four-pole radial magnetic bearing with permanent magnets under consideration. The four-pole magnetic bearing consists of a stator, rotor, four coils and four permanent magnets. The stator and rotor are made of M400-50A silicon steel sheets to significantly reduce eddy currents induced due to the rotor rotation and the change in the magnetic flux due to the control current. The B–H curve of the M400-50A silicon steel measured with a closed magnetic circuit method is presented in Figure 2.

Due to the significant limitation of the eddy currents, the finite-element model was used to perform the magnetostatic simulations. The air gap between the stator and rotor equals 300 µm. The stator has four wounded poles, two for the *y*-axis and another two for the *x*-axis. The turn number of each winding equals 100. The cross-sectional area of the winding slot equals 120 mm^2^. The current density inside the windings equals approx. 5.6 A/mm^2^, and taking into account the slot filling factor equals 70%. Four permanent NdFeB magnets (N38, *B_r_* = 1.23 T, *H_c_* = 63,800 A/m) are used to produce the bias flux. In Table 1, the main geometrical parameters of the magnetic bearing with permanent magnets are listed.

**Figure 3:**
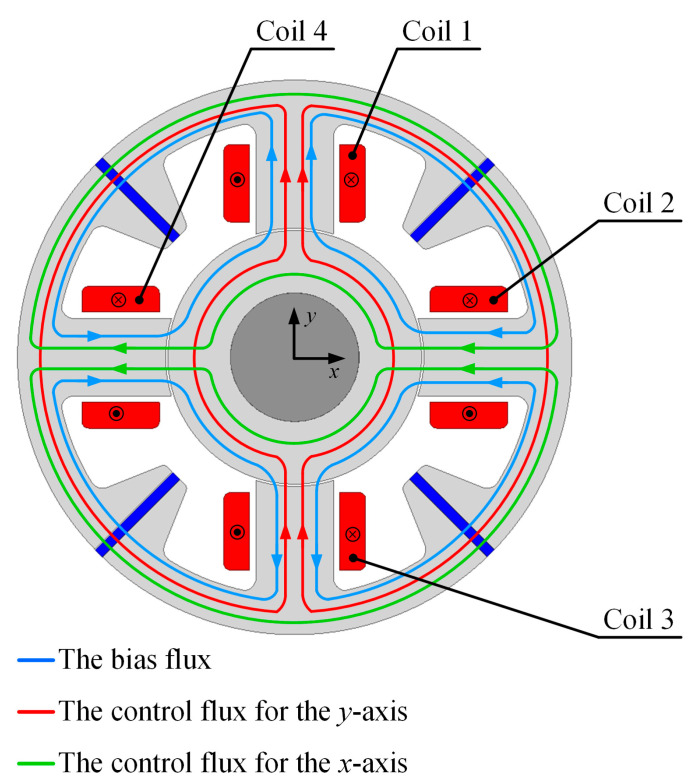
Magnetic flux paths inside the magnetic bearing.

Figure 3 presents paths of the magnetic flux produced by the permanent magnets and windings.

Four permanent magnets produce the bias flux, which determines the operating point of the magnetic circuit. Their geometrical parameters were chosen intentionally to obtain the magnetic flux density (approx.0.8 T) inside the air gap. The bias flux passes through the poles, rotor and stator yoke. For the rotor central position, the value of the magnetic flux density in the air gap is the same as for all the poles, which causes the generated magnetic forces along the *x*- and *y*-axis to be equal to zero. Meanwhile, four coils produce the control flux, which allows for changing the magnetic force acting on the rotor along the *x*- and *y*-axis. Coils 1 and 3 are connected in series, so that for the positive control current, the magnetic flux density inside the first pole increases, while in the third pole it decreases. Therefore, the positive control current *i_y_* produces the positive value of magnetic force *F_y_* along the *y*-axis. Similarly, coils 2 and 4 are connected in series, and the positive value of the control current *i_x_* also generates the positive value of the magnetic force *F_x_* along the *x*-axis.

## 3. Magnetic Simulation of the Magnetic Bearing

**Figure 4:**
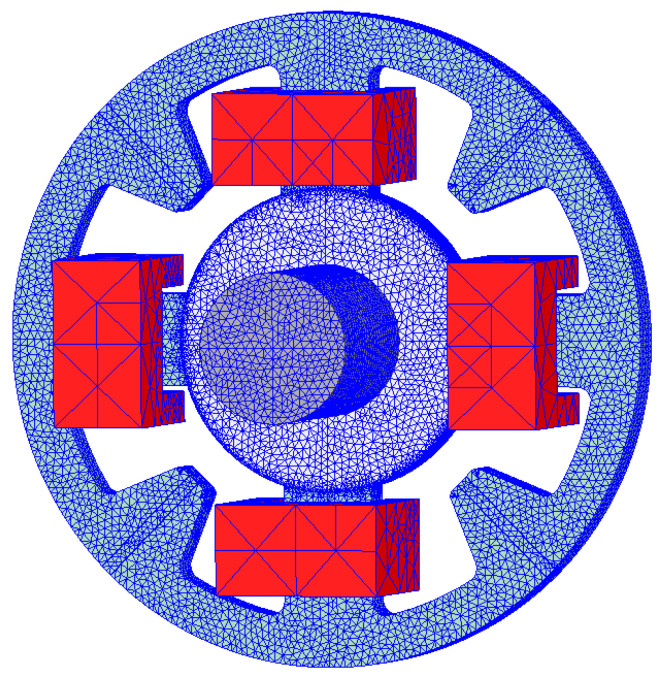
The finite-element model discretization for the four-pole magnetic bearing.

Figure 4 depicts a three-dimensional finite-element model (3D FEM) prepared in the Ansys Maxwell 3D software ver. 2021 R1. To limit the calculation time, only half of the magnetic bearing geometry was simulated. The simulation area was limited by the cuboid, whose faces were distanced from the stator and rotor by 50 mm, except for the symmetry plane that was in the middle of the stator length. The zero Dirichlet boundary condition was assumed on the outer faces of the simulation model, while on the symmetry plane, the zero Neumann boundary condition was set. The size of the tetrahedral elements was set manually to obtain a fine mesh. Special care was taken to discretize the air gap. The total number of elements in the simulation model was approx. 330,000.

The Ansys Maxwell 3D ver. 2021 R1 software uses an implementation of the two vector and scalar potentials $\vec{T}-\Omega$ for solving the electromagnetic field. The basic equations for the $\vec{T}-\Omega$ method are as follows:

(1) $$\nabla \cdot \mu \left(\vec{T}-\nabla \Omega \right)=0$$

(2) $$\nabla \times (\nabla \times \vec{T})=-\gamma \mu \frac{\partial }{\partial t}\left(\vec{\nabla T}-\Omega \right)$$

where *µ* is the permeability and *σ* is the conductivity.

The current vector potential $\vec{T}$ is defined by its circulation as follows:

(3) $$\vec{J}=\nabla \times \vec{T}$$

While the magnetic scalar potential *Ω* is defined with its gradient as follows:

(4) $$\vec{H}=\vec{T}-\nabla \Omega$$

where $\vec{H}$ is the magnetic field intensity.

The simulation of the field analysis was used to calculate the magnetic force and the linkage flux of the windings. The magnetic force along the *x*- and *y*-axis was calculated from the method of virtual work as follows:

(5) $$F_{x}=\frac{\partial W_{co}}{\partial x}$$

(6) $$F_{y}=\frac{\partial W_{co}}{\partial y}$$

where *W_co_* denotes the magnetic coenergy.

The flux linkages of the field excitation windings *Ψ_y_* and *Ψ_x_* were calculated as the sum of the flux linked with coils as follows:

(7) $$\Psi_{x}=\Psi_{1}+\Psi_{3}=N{\phi \;}_{1}+N{\phi \;}_{3}$$

(8) $$\Psi_{y}=\Psi_{2}+\Psi_{4}=N{\phi \;}_{2}+N{\phi \;}_{4}\;$$

where $\Psi_{1}$, $\Psi_{2}$, $\Psi_{3}$ and $\Psi_{4}$ are the flux linkage of the first, second, third and fourth coil, respectively. The letter *N* denotes the number of winding turns. The magnetic flux *ϕ_k_* linked with one turn of a coil wire is calculated as follows:

(9) $$\phi_{k}=\iint_{S_{k}}^{\;}\vec{B}\cdot d{\vec{S}}_{k}$$

where $\vec{B}$ is the magnetic field density inside the *k*^th^ coil turn and *S_k_* is the area bounded by the turn.

**Figure 5:**
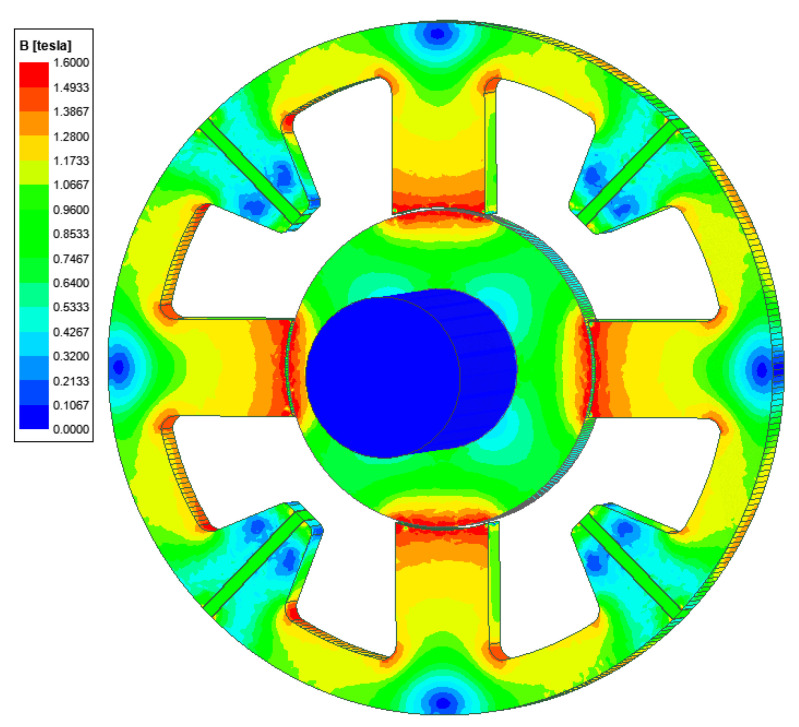
The magnetic flux density distribution for the rotor central position and lack of the control currents.

Figure 5 presents the magnetic flux distribution for the central position of the rotor and the lack of control currents. It can be seen that the magnetic flux density in the significant part of the magnetic circuit was equal to 1.0 T.

**Figure 6:**
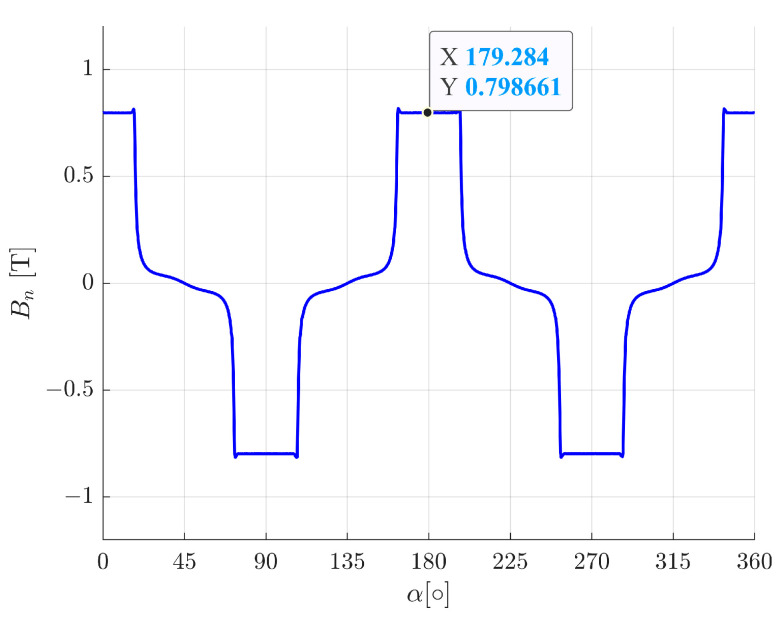
The normal component to the rotor surface of the magnetic flux density in the air gap for the rotor central position and lack of control currents.

Figure 6 presents the values of the normal component (to the stator surface) of the magnetic flux density in the air gap for the rotor central position and the lack of a control current.

**Figure 7:**
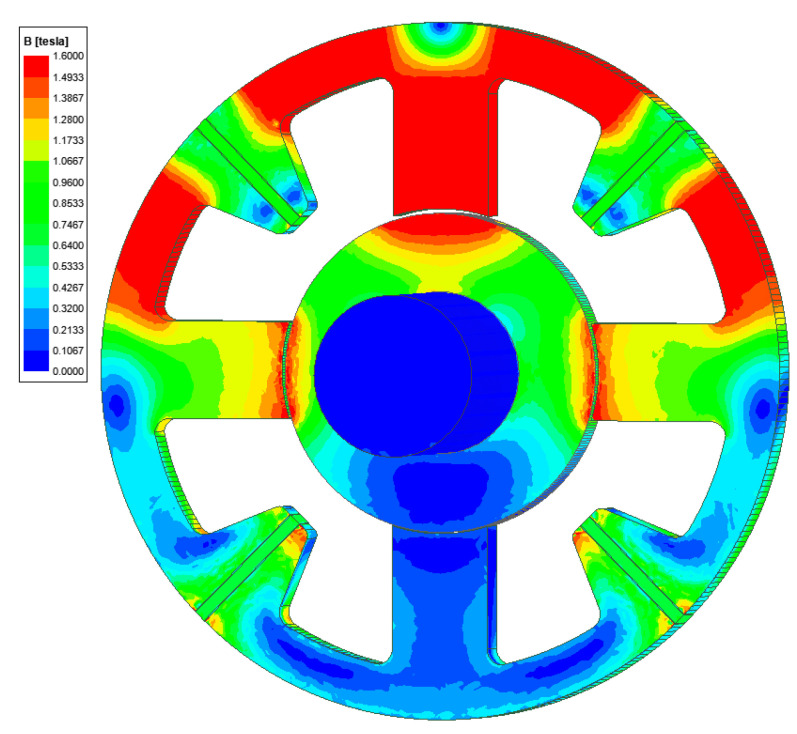
The magnetic flux density distribution for the position of the rotor at *y* = −0.2 mm and the control current of *i_y_* = 4.6 A.

Figure 7 presents the magnetic flux density map for the position of the rotor at *y* = −0.2 mm and a control current intensity of *i_y_* = 4.6 A. For this operation condition, the magnetic bearing generated a magnetic force of 64.54 N, which is sufficient for lifting the rotor.

**Figure 8:**
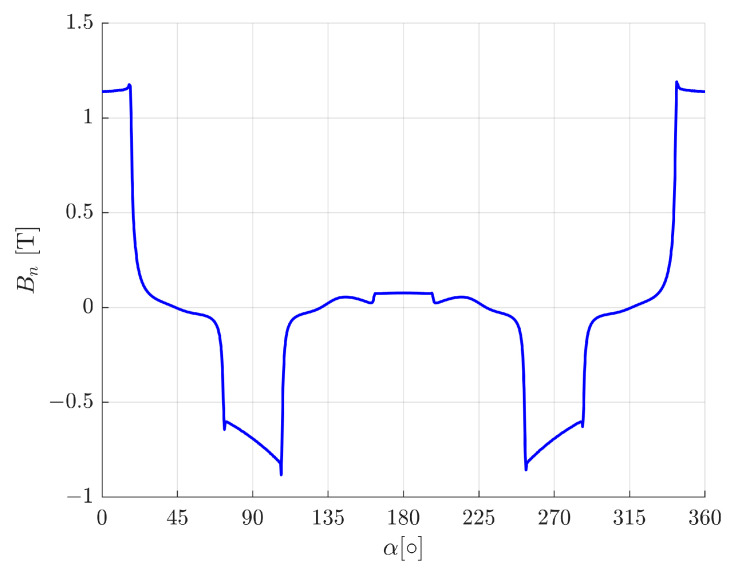
The normal (to the rotor surface) component of the magnetic flux density, in the air gap, for the rotor position of *y* = −0.2 mm and the control current intensity of *i_y_* = 4.6 A.

Figure 8 presents the normal component (to the stator surface) of the magnetic flux density in the air gap for the rotor position of −0.2 mm and the control current intensity of 4.6 A. It can be seen that the magnetic flux density in the air gap, under the lower pole, amounted to almost zero. For such conditions, the magnetic bearing generated a maximal initial force.

**Figure 9:**
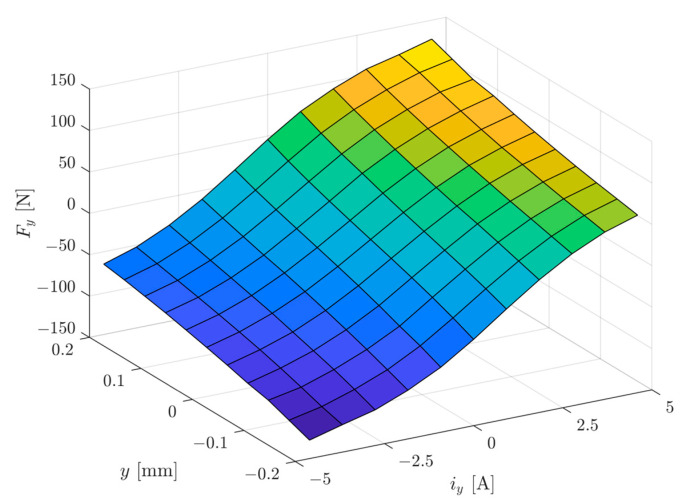
The magnetic force *F_y_* as a function of the rotor position of *y* ∈ (−0.2 mm, 0.2 mm) and the control current of *i_y_* ∈ (−4.6 A, 4.6 A).

Figure 9 presents the magnetic force *F_y_* as a function of the rotor position of *y* ∈ (−0.2 mm, 0.2 mm) and the control current of *i_y_* ∈ (−4.6 A, 4.6 A). It can be seen that the maximal force *F_max_* (calculated for the position of *y* = 0 mm and the control current of *i_y_* = 4.6 A) was equal to 90.05 N, while the initial force *F_init_* (calculated for the position of *y* = −0.2 mm and the control current of *i_y_* = 4.6 A) amounted 64.54 N; therefore, this magnetic bearing can lift a rotor with a weight of about 2 kg.

**Figure 10:**
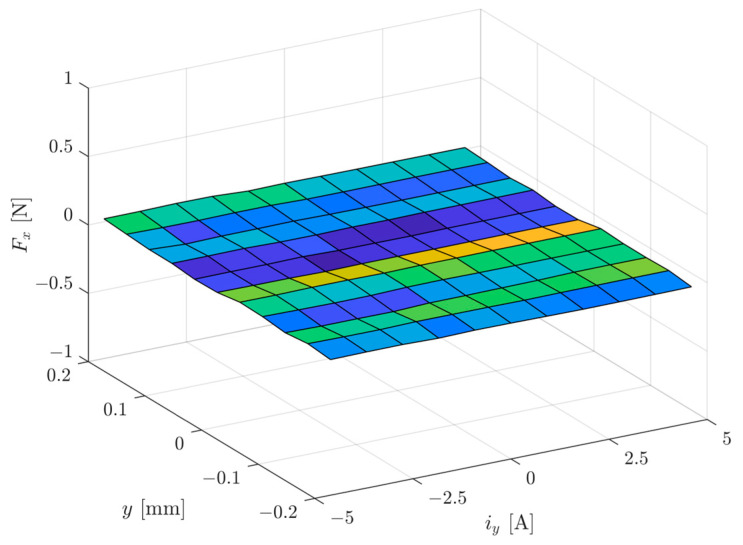
The magnetic force *F_x_* as a function of the rotor position of *y* ∈ (−0.2 mm, 0.2 mm) and control current of *i_y_* ∈ (−4.6 A, 4.6 A).

Figure 10 presents the magnetic force *F_x_* as a function of the rotor position of *y* ∈ (−0.2 mm, 0.2 mm) and the control current of *i_y_* ∈ (−4.6 A, 4.6 A). It can be seen that the presented magnetic bearing has no cross-coupling between axes.

**Figure 11:**
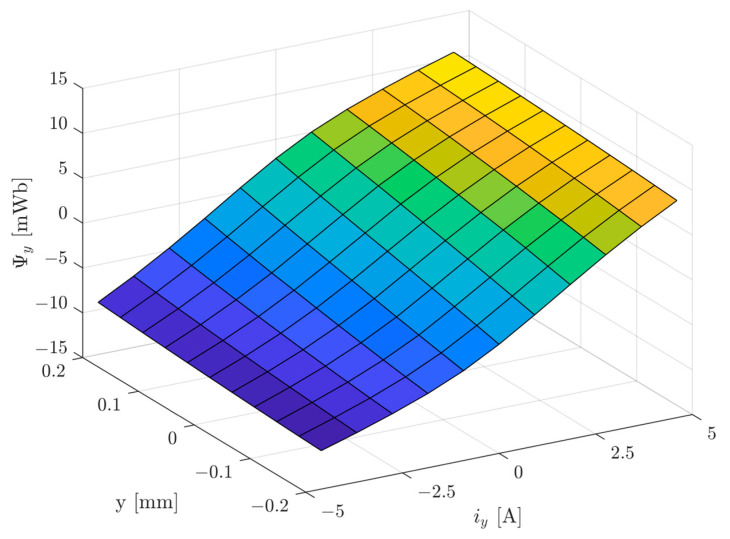
The linkage flux of the control winding *Ψ_y_* for the *y*-axis as a function of the rotor position of *y* ∈ (−0.2 mm, 0.2 mm) and the control current of *i_y_* ∈ (−4.6 A, 4.6 A).

Figure 11 presents the linkage flux *Ψ_y_* of the control winding for the *y*-axis as a function of the rotor position of *y* ∈ (−0.2 mm, 0.2 mm) and control current of *i_y_* ∈ (−4.6 A, 4.6 A). The control winding consists of two coils connected in series.

The characteristics for the *x*-axis are identical to those for the *y*-axis; therefore, they are not presented in this paper.

The presented simulation model was used to calculate the parameters of the magnetic bearing. The characteristic of the magnetic force was used to determine the maximal force, *F_max_*, as well as the initial force *F_init_* and the current *k_i_* and position *k_s_* stiffness, while the linkage flux was used to obtain the dynamic inductance *L_d_* and the velocity-induced voltage *e_v_* [19]. In Table 2, the parameters of the magnetic bearing are listed. All the parameters have the same value for both axes; therefore, they are listed only for the *y*-axis.

The dynamic inductance as well as the velocity-induced voltage were calculated based on the linkage flux according to these expressions given in [19]:

(10) $$L_{dx}=\frac{\partial \Psi_{x}\left(i_{x},x\right)}{\partial i_{x}}$$

(11) $$L_{dy}=\frac{\partial \Psi_{y}\left(i_{y},y\right)}{\partial i_{y}}$$

(12) $$e_{vx}=\frac{\partial \Psi_{x}\left(i_{x},x\right)}{\partial_{x}}$$

(13) $$e_{vy}=\frac{\partial \Psi_{y}\left(i_{y},y\right)}{\partial_{y}}$$

Two constructions of a six-pole magnetic bearings with permanent magnets are described in [19]. They have a similar geometry to the construction presented in this research. Nevertheless, the four-pole magnetic bearing presented in this paper has better parameters; in particular, it has a higher value of the current stiffness coefficient and a beneficial lower value of the position stiffness.

## 4. Transient Time Simulations

**Figure 12:**
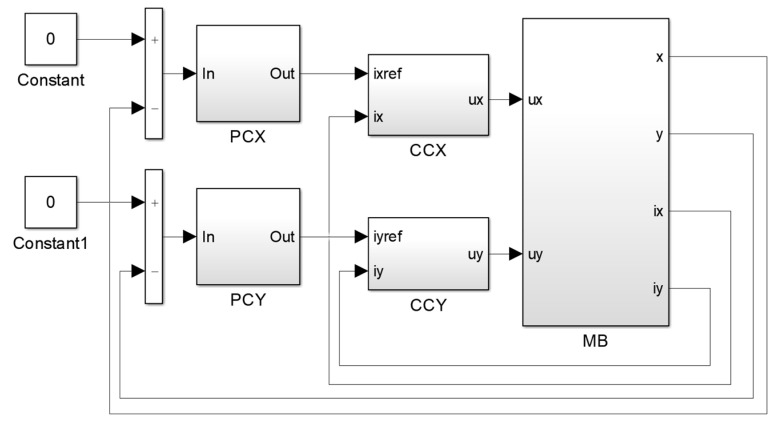
Implementation of the control system in MATLAB/Simulink software (abbreviations: PCX—the position controller in the *x*-axis, PCY—the position controller in the *y*-axis, CCX—the current controller in the *x*-axis, CCY—the current controller in the *y*-axis, MB—the magnetic bearing).

Transient time simulations were carried out using the field-circuit indirectly coupled finite-element model prepared in the MATLAB/Simulink software ver. R2022b. The simulation model consisted of two components: the results obtained from the finite-element model implemented as look-up tables and equations that describe the electrical and mechanical behavior of the magnetic bearing. The magnetic bearing is an unstable device; therefore, the levitation of the rotor required a control system. There are various implementations of control systems, but the most commonly used control systems consist of position and current controllers. Position controllers along the *x*- and *y*-axis determine the control currents *i_x_* and *i_y_* based on the signals from the position sensors, whereas the current controllers regulate the currents that flow through the windings. Figure 12 presents an implementation of the control system in the MATLAB/Simulink software, where position controllers (abbreviations: PCX and PCY) were implemented as discrete PID controllers and current controllers (abbreviations: CCX and CCY) were implemented as discrete PI controllers.

The discrete position controllers were implemented according to the following equation:

(14) $$G_{PID}\left(z\right)=K_{P}+K_{I}\frac{T_{s}}{z-1}+K_{D}\frac{N}{1+\frac{T_{s}N}{z-1}}$$

where *K_P_*, *K_I_* and *K_D_* are the parameters of the PID controller. *T_s_* denotes the sampling time, and *N* indicates the filter coefficient of the derivative part. All controllers contain an integrator anti-windup circuit [20].

The paper [21] describes a method for the calculation of the position controllers’ parameters based on the stiffness *k* and damping *c* coefficients. The same method was used for this work. Transient time simulations were carried out for two sets of the stiffness coefficient *k* and the damping coefficient *c*. For the first set, the stiffness coefficient *k* was equal to 60,000 N/m and the damping coefficient *c* was equal to 550 Ns/m. For the second set, the stiffness coefficient *k* was equal to 90,000 N/m and the damping coefficient *c* was equal to 850 Ns/m. The values of these parameters were taken arbitrarily because they depend on the specific application of the magnetic bearing. However, an increase in the stiffness *k* and damping *c* coefficients demands an improvement in the PID controller parameters, and that requires a higher sampling rate of the whole control system and a better quality of the control signals.

Due to the symmetry of the magnetic circuit, the parameters of the position controllers for both axes were the same. In Table 3, the values of the position controller parameters for the two sets are listed.

**Figure 13:**
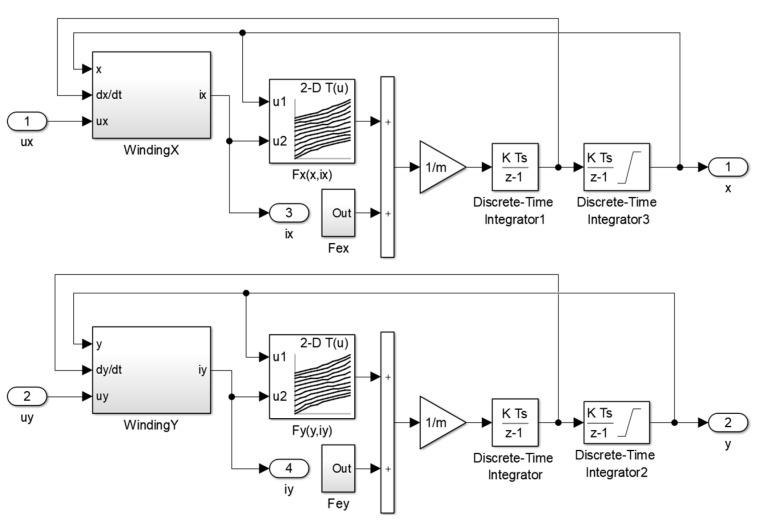
An implementation of the mechanical component of the simulation model.

Figure 13 presents an implementation of the mechanical component for the field-circuit indirectly coupled finite-element model of the magnetic bearing, which was based on the following equations:

(15) $$m\frac{d^{2}x}{dt^{2}}=F_{x}\left(x,i_{x}\right)+F_{ex}$$

(16) $$m\frac{d^{2}y}{dt^{2}}=F_{y}\left(y,i_{y}\right)-mg+F_{ey}$$

where *F_x_*(*x*, *i_x_*) and *F_y_*(*y*, *i_y_*) are the magnetic forces acting along the *x*- and *y*-axis, respectively, *m* denotes the mass of the rotor, *g* indicates gravitational acceleration and *F_ex_* and *F_ey_* are external forces acting on the rotor along the *x*- and *y*-axis, respectively.

**Figure 14:**
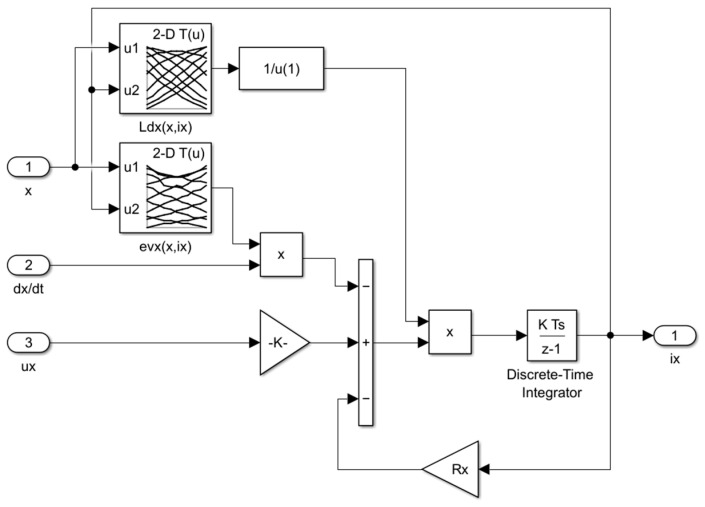
An implementation of the electric part for the *x*-axis of the simulation model.

Figure 14 presents an implementation of the electrical component for the *y*-axis of the field-circuit indirectly coupled finite-element model of the magnetic bearing, which was based on the following equations:

(17) $$\frac{di_{x}}{dt}=\frac{1}{L_{dx}\left(x,{\;i}_{x}\right)}\left(u_{x}-R_{x}i_{x}-e_{vx}\left(x,i_{x}\right)\frac{dx}{dt}\right)$$

(18) $$\frac{di_{y}}{dt}=\frac{1}{L_{dy}\left(y,{\;i}_{y}\right)}\left(u_{y}-R_{y}i_{y}-e_{vy}\left(y,i_{y}\right)\frac{dy}{dt}\right)$$

where *u_x_* and *u_y_* are the supply voltages for the *x*- and *y*-axis, respectively, and *R_x_* and *R_y_* indicate the resistance of the control windings for the *x*- and *y*-axis, respectively.

The constant parameters of the dynamic simulation model were as follows: the mass of the rotor *m* was equal to 1.54 kg and resistances of the windings *R_x_* and *R_y_* were equal to 0.3 Ω.

**Figure 15:**
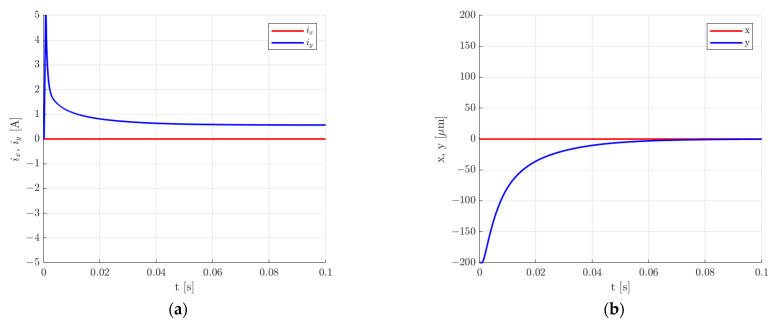
Time responses of the control current (**a**) and the rotor position (**b**) for the rotor lifting from the starting position for the first set of the control system parameters.

**Figure 16:**
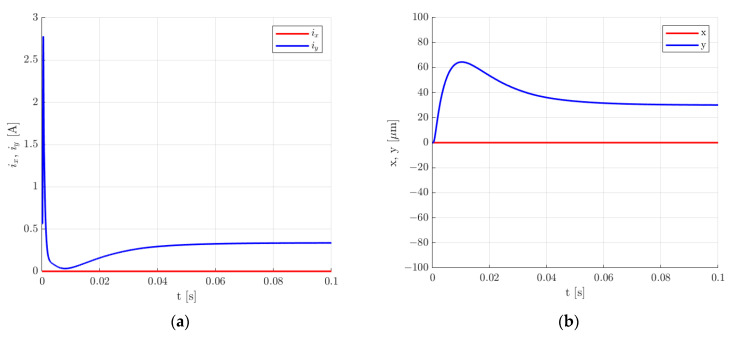
Time responses of the control current (**a**) and the rotor position (**b**) for the step change of 30 µm in the rotor position along the *y*-axis for the first set of the control system parameters.

**Figure 17:**
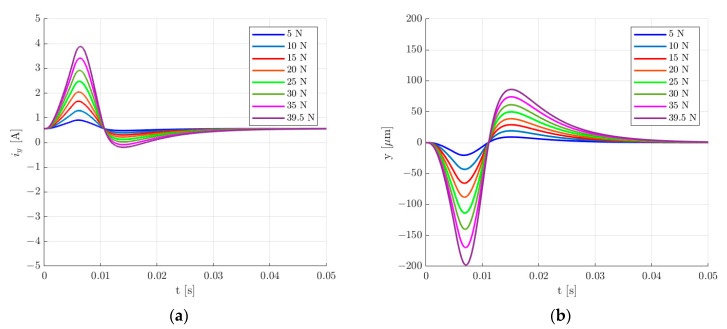
Time responses of the control current (**a**) and the rotor position (**b**) for the change in the rotor position under an external impact force applied along the *y*-axis for the first set of the control system parameters.

**Figure 18:**
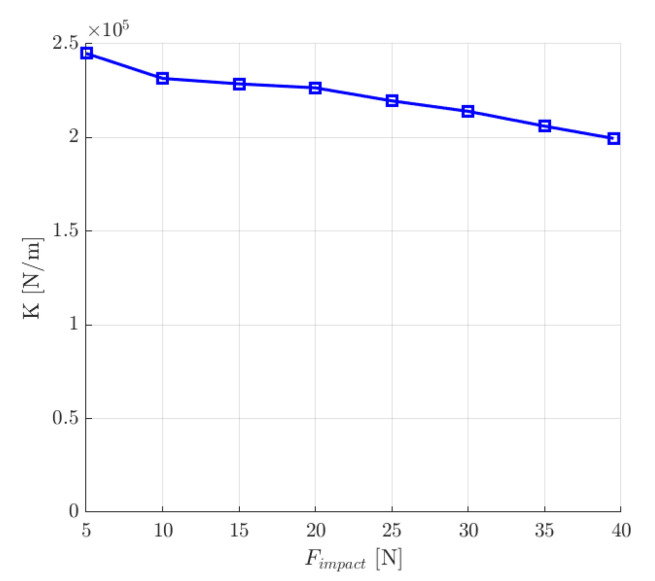
The dynamic stiffness *K* as a function of the external impact force *F_impact._*.

The presented simulation model was used to calculate the transient response for the rotor lifting from the starting position, a step change of 30 µm in the rotor position along the *y*-axis and the change in the rotor position under an external impact force applied along the *y*-axis for the two sets of control system parameters. Figure 15, Figure 16, Figure 17 and Figure 18 present the results for the first set of the control system parameters. Figure 14 indicates that the settling time for the rotor lifting to the equilibrium position was equal to 40 ms. There was no overshooting, and the control current required for the rotor levitation was equal to 0.566 A.

The 30 µm step change in the rotor position along the *y*-axis caused an overshoot equal to 214.86%. The settling time *t_s_* for the 5% error band was equal to 61 ms. The change in the rotor position caused a decrease in the value of the control current to 0.336 A.

Various values of the external impact force were applied to determine the maximal force that causes the maximal allowed movement of the rotor. For the maximal peak value of 39.5 N, the rotor deviated from the equilibrium position by approximately 198 µm (Figure 17a). Therefore, based on the simulation results, the maximal value of the external impact force that can be applied to the rotor of the analyzed magnetic bearing with the first set of the control system parameters equals 39.5 N.

This numerical experiment also allowed for the determination of the dynamic stiffness *K* of the magnetic bearing system, which was calculated from the following expression [12]:

(19) $$K=\frac{F_{impact}}{p_{max}}$$

where *F_impact_* is the value of the external impact force and *p_max_* is the maximum deviation from the equilibrium position.

As can be seen from Figure 18, the value of the dynamic stiffness decreased with the increase in the impact force *F_imapact_*.

**Figure 19:**
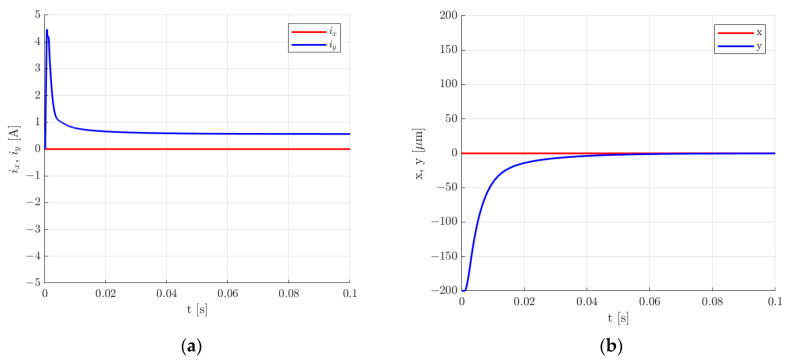
Time responses of the control current (**a**) and the rotor position (**b**) for the rotor lifting from the starting position for the second set of the control system parameters.

**Figure 20:**
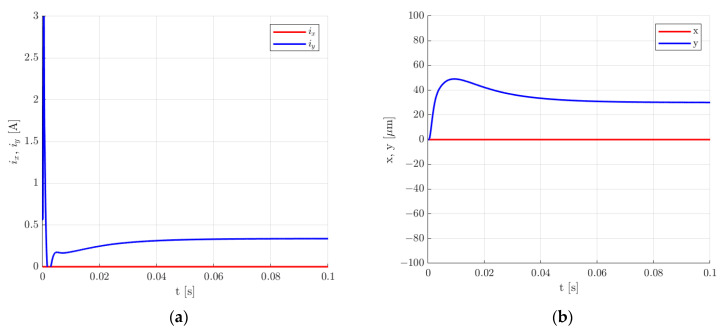
Time responses of the control current (**a**) and the rotor position (**b**) for the step change of 30 µm in the rotor position along the *y*-axis for the second set of the control system parameters.

**Figure 21:**
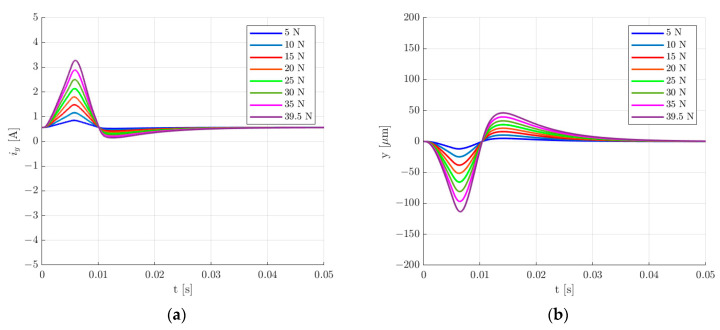
Time responses of the control current (**a**) and the rotor position (**b**) for the change in the rotor position under an external impact force applied along the *y*-axis for the second set of the control system parameters.

**Figure 22:**
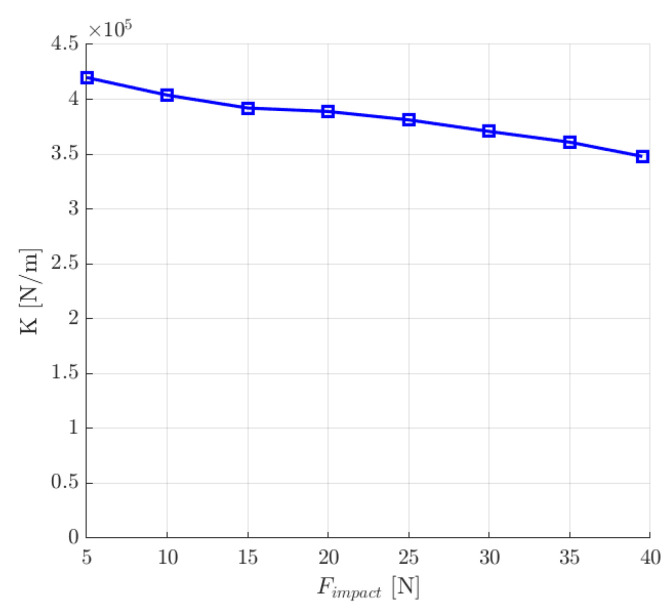
The dynamic stiffness *K* as a function of the external impact force.

Figure 19, Figure 20, Figure 21 and Figure 22 present the results for the second set of control system parameters. Figure 19 indicates that the settling time for the rotor lifting to the equilibrium position was shorter in comparison to the previous set of control system parameters and was equal to 24.5 ms. Similarly to the previous set of control system parameters, there was no overshooting, and the control current required for the rotor levitation was equal to 0.566 A.

For the second set of control system parameters, the 30 µm step change in the rotor position along the *y*-axis also caused overshooting, but in that case, the overshooting was equal to 163.57%. Additionally, the settling time *t_s_* for the 5% error band was smaller and equal to 53 ms. The value of the control current was the same as for the first set of control system parameters and equal to 0.336 A.

To compare the response of the rotor position under an external impact force, the same values of the impact force were applied along the *y*-axis as in the previous set of control system parameters. It can be seen that for the second set of control system parameters, deviation from the equilibrium position was smaller (Figure 21b). Therefore, the values of the dynamic stiffness *K* were higher for the magnetic bearing system with the second set of the control system parameters (Figure 22). Similarly to the first set of control system parameters, the value of the dynamic stiffness *K* decreased with an increase in the impact force *F_impact_*.

**Figure 23:**
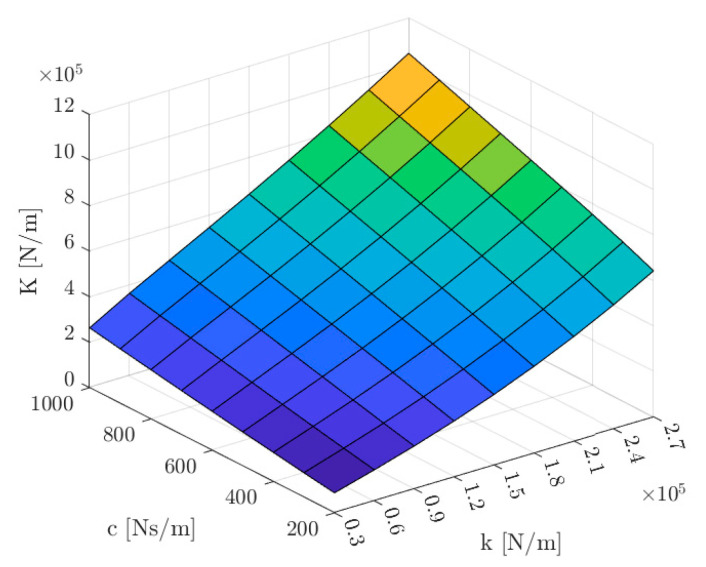
The dynamic stiffness *K* as a function of the stiffness coefficient *k* and the damping coefficient *c* for the external impact force *F_impact_* equal to 10 N.

In Figure 23, the dynamic stiffness *K* is presented as a function of the stiffness *k* and damping c coefficients for the external impact force *F_impact_* equal to 10 N. It can be seen that in the analyzed range, the dynamic stiffness *K* only increased.

## 5. Conclusions

This paper presents a design of a four-pole radial magnetic bearing with permanent magnets installed in the stator yoke. The rated parameters as well as the values of the magnetic forces, dynamic inductances and velocity-induced voltages as the function of the control currents and rotor positions were obtained from the 3D finite-element model. The proposed magnetic bearing has better parameters than other reported magnetic bearings with permanent magnets with comparable geometrical parameters. The calculated physical quantities were implemented in the presented field-circuit indirectly coupled finite-element model. The dynamic simulation model was used to calculate the waveforms of the essential dynamic responses, like the lifting of the rotor, the step change in the rotor position and the change in the rotor position under an external impact force. The simulation results demonstrated that an increase in the stiffness coefficient *k* or an increase in the damping coefficient *c* causes an increase in the value of the dynamic stiffness. The presented simulations confirmed that the proposed magnetic bearing construction with permanent magnets can successfully levitate the rotor.

## Figures and Tables

**Table 1:** Main geometrical parameters of the magnetic bearing with permanent magnets.

Parameter	Value
Outer diameter of the stator, *d_so_*	86.0 mm
Inner diameter of the stator, *d_si_*	40.0 mm
Outer diameter of the rotor, *d_ro_*	39.6 mm
Stator length, *l_s_*	10.0 mm
Width of the pole, *w_p_*	12.0 mm
Width of the permanent magnet, *w_pm_*	1.5 mm
Permanent magnet thickness, *t_pm_*	17.0 mm

**Table 2:** Rated parameters for the magnetic bearing with permanent magnets.

Parameter	Value
Current stiffness, *k_iy_*	26.81 N/A
Position stiffness, *k_sy_*	198,453.52 N/m
Maximal force, *F_maxy_*	90.05 N
Initial force, *F_inity_*	64.54 N
Dynamic inductance, *L_dy_*	2.65 mH
Velocity-induced voltage, *e_vy_*	13.58 Vs/m

**Table 3:** Parameters of the position controller.

Parameter	Values for *k* = 60,000 N/m and *c* = 550 Ns/m (Set 1)	Values for *k* = 90,000 N/m and *c* = 850 Ns/m (Set 2)
Proportional gain, *K_P_*	13,689.50	18,423.66
Derivative gain, *K_D_*	31.85	45.59
Integral gain, *K_I_*	286,846.12	526,969.97
Derivate filter divisor, *N*	3000	3000
Sampling time, *T_s_*	100 µs	100 µs

## Data Availability

Dataset available on request from the authors.
